# Durability of the Treatment Effects of an 8-Week Self-administered Home-Based Virtual Reality Program for Chronic Low Back Pain: 6-Month Follow-up Study of a Randomized Clinical Trial

**DOI:** 10.2196/37480

**Published:** 2022-05-25

**Authors:** Laura Garcia, Brandon Birckhead, Parthasarathy Krishnamurthy, Ian Mackey, Josh Sackman, Vafi Salmasi, Robert Louis, Carina Castro, Roselani Maddox, Todd Maddox, Beth D Darnall

**Affiliations:** 1 AppliedVR Van Nuys, CA United States; 2 Johns Hopkins School of Medicine Baltimore, MD United States; 3 University of Houston Houston, TX United States; 4 Stanford University School of Medicine Palo Alto, CA United States; 5 Hoag Memorial Hospital Newport Beach, CA United States; 6 Stanford School of Medicine Palo Alto, CA United States

**Keywords:** behavioral health, chronic low back pain, treatment, virtual reality

## Abstract

**Background:**

We previously reported the efficacy of an 8-week home-based therapeutic immersive virtual reality (VR) program in a double-blind randomized placebo-controlled study. Community-based adults with self-reported chronic low back pain were randomized 1:1 to receive either (1) a 56-day immersive therapeutic pain relief skills VR program (EaseVRx) or (2) a 56-day sham VR program. Immediate posttreatment results revealed the superiority of therapeutic VR over sham VR for reducing pain intensity; pain-related interference with activity, mood, and stress (but not sleep); physical function; and sleep disturbance. At 3 months posttreatment, therapeutic VR maintained superiority for reducing pain intensity and pain-related interference with activity, stress, and sleep (new finding).

**Objective:**

This study assessed between-group and within-group treatment effects 6 months posttreatment to determine the extended efficacy, magnitude of efficacy, and clinical importance of home-based therapeutic VR.

**Methods:**

E-surveys were deployed at pretreatment, end-of-treatment, and posttreatment months 1, 2, 3, and 6. Self-reported data for 188 participants were analyzed in a mixed-model framework using a marginal model to allow for correlated responses across the repeated measures. Primary outcomes were pain intensity and pain-related interference with activity, mood, stress, and sleep at 6 months posttreatment. Secondary outcomes were Patient-Reported Outcome Measurement Information System (PROMIS) sleep disturbance and physical function.

**Results:**

Therapeutic VR maintained significant and clinically meaningful effects 6 months posttreatment and remained superior to sham VR for reducing pain intensity and pain-related interference with activity, stress, and sleep (*d_s_*=0.44-0.54; *P*<.003). Between-group comparisons for physical function and sleep disturbance showed superiority of EaseVRx over sham VR (*d_s_*=0.34; *P*=.02 and *d_s_*=0.46; *P*<.001, respectively). Participants were encouraged to contact study staff with any problems experienced during treatment; however, no participants contacted study staff to report adverse events of any type, including nausea and motion sickness.

**Conclusions:**

Our 8-week home-based VR pain management program caused important reductions in pain intensity and interference up to 6 months after treatment. Additional studies are needed in diverse samples.

**Trial Registration:**

ClinicalTrials.gov NCT04415177; https://clinicaltrials.gov/ct2/show/NCT04415177

**International Registered Report Identifier (IRRID):**

RR2-10.2196/25291

## Introduction

Chronic low back pain (CLBP) is the most common persistent pain condition worldwide, and multiple barriers impede patient access to timely and effective care. Innovations in digital therapeutics, such as immersive virtual reality (VR), offer the promise of home-based care, broad availability of treatment, and the potential to address the needs of underserved populations with CLBP.

Immersive VR is an evidence-based analgesic for acute low back pain [1], procedural low back pain [2], and CLBP [3,4]. Many VR treatments for CLBP involve rehabilitation exercise and require therapist guidance [5]. However, recent chronic pain research has investigated fully self-administered VR programs that require no clinician contact or guided movement exercises. Such programs closely mirror the content delivered in pain self-management or evidence-based psychological treatments for chronic pain.

In 2 randomized trials [3,4], we evaluated the effectiveness of a therapeutic VR program that incorporated multiple pain management modalities delivered via brief daily VR sessions. The first trial compared a 3-week skills-based VR program to the same therapeutic content delivered in audio-only format in 79 individuals with CLBP or fibromyalgia [3]. Posttreatment results revealed that the immersive VR modality was superior to the audio-only modality for reducing pain intensity and pain-related interference with activity, mood, sleep, and stress.

The second trial was a double-blind, randomized, placebo-controlled comparison of 8-week self-administered behavioral skills-based VR (EaseVRx; AppliedVR) with sham VR in 188 adults with CLBP. The 8-week sham VR program consisted of 2D placebo content involving nonimmersive nature scenes and neutral music (no skills training or pain education) [4,6]. Both treatments were delivered via the same commercial VR headsets and involved brief daily treatment sessions. Intention-to-treat analyses revealed benefits in both treatment groups and the superiority of therapeutic VR over sham VR for reducing pain intensity and pain-related interference with activity, stress, and mood, as well as sleep disturbance, with large effect sizes ranging from 1.17 to 1.3 (moderate to substantial clinical importance). On comparing the groups, a greater proportion of participants in the EaseVRx group achieved ≥30% reduction in pain intensity, and 46% of EaseVRx participants achieved ≥50% reduction in pain [4]. At 3 months, EaseVRx showed significant superiority over sham VR for reducing pain intensity and pain-related interference (activity, stress, and sleep [new finding]), with moderate to large effect sizes (0.56-0.88) exceeding the thresholds for clinical meaningfulness [7].

This study extended the results of this same study sample (N=188) [4,7] to 6 months posttreatment to evaluate further the durability of VR treatment. This study also included outcomes for participant blinding and treatment group unmasking at 6 months posttreatment. Finally, we investigated whether therapeutic VR engagement differs by socioeconomic status (SES), using a variable comprised of education level and annual household income.

## Methods

### Study Design

This 6-month follow-up study used a single-cohort, placebo-controlled, randomized clinical trial protocol [6]. The study involved an online national convenience sample of 188 community-based adults with self-reported CLBP.

The 6-month posttreatment data collection was completed in April 2021. Participants were instructed to return their VR headsets within 5 days of completing their 56-day treatment period (postage-paid packaging provided). Any headset returned after this 5-day shipment period was considered a late return.

This report contains participant-reported data from e-surveys deployed at pretreatment, end-of-treatment (day 56), and posttreatment months 1, 2, 3, and 6 for the primary outcomes (average pain intensity and pain-related interference with activity, mood, sleep, and stress) and the two secondary outcomes of sleep disturbance and physical function that demonstrated immediate effects after treatment.

Detailed information of the methods and interventions is provided in the study protocol [6].

### Ethical Considerations

The Western Copernicus Group Institutional Review Board (Puyallup, WA) approved the study protocol in July 2020 (number: 1286465). Eligible individuals were enrolled after signing an eConsent form.

### Participants

**Figure 1:**
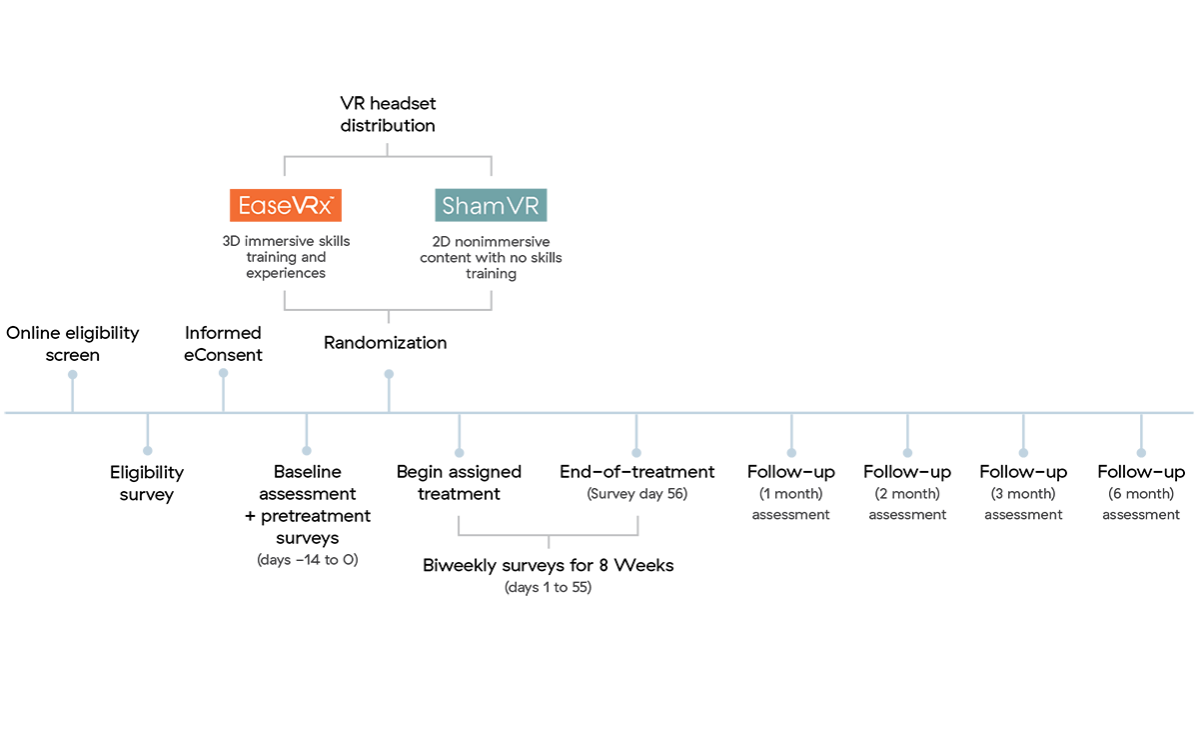
Participant activities. VR: virtual reality.

Individuals with CLBP were recruited nationally through Facebook and Google online advertisements, chronic pain organizations, and professional colleagues. Advertisements directed individuals to the study website for information, and they were invited to complete an online eligibility form (see Textbox 1 for the inclusion/exclusion criteria). Figure 1 displays the participant study activities.

**Study inclusion and exclusion criteria.**

**Inclusion criteria **

1. Men and women aged 18-85 years 
2. Self-reported diagnosis of chronic low back pain without radicular symptoms
3. Chronic low back pain duration ≥6 months 
4. Average pain intensity of ≥4 for the past month (0-10 numeric pain rating scale)
5. English fluency
6. Willing to comply with study procedures and restrictions 
7. Wi-Fi access
8. Implicit de facto internet and computer literacy

**Exclusion criteria**

1. Gross cognitive impairment
2. Current or prior diagnosis of epilepsy, seizure disorder, dementia, migraines, or other neurological diseases that may prevent the use of virtual reality (VR) or predispose to adverse effects
3. Medical condition predisposing to nausea or dizziness 
4. Hypersensitivity to flashing lights or motion 
5. No stereoscopic vision or severe hearing impairment 
6. Injury to the eyes, face, or neck that impedes comfortable use of a VR headset
7. Cancer-related pain
8. Depressive symptoms ≥2 on the Patient Health Questionnaire-2 (PHQ-2) depression screen
9. Previous use of EaseVRx for pain 
10. Current or recent completion of participation (past 2 months) in any interventional research study
11. Currently pregnant or planning to become pregnant during the study period
12. Currently working at or having an immediate family member who works for a digital health company or pharmaceutical company that provides treatment for acute or chronic pain

### Randomization and Participant Blinding

Enrolled participants completed a baseline survey battery and a pain survey that was readministered 3 times during the 2-week pretreatment period. These surveys were averaged to create a pretreatment pain value; completion of at least two surveys was required to progress to the treatment phase. The conduct of the study was entirely remote.

E-randomization was applied 1:1 without blocking and with participants allocated to either (1) a 56-day skills-based pain relief VR program (EaseVRx) or (2) a 56-day VR control condition (sham VR). Study participants understood they would be assigned to 1 of 2 VR treatments, but did not know that 1 treatment was sham. Participants remained blinded to their group assignment until all data were collected 6 months posttreatment. Participants were then informed that the study involved random assignment to VR with or without active treatment for chronic pain and were asked which program they believed they received. The statistician performed blinded analysis for the 56-day end-of-treatment results [4] and was unblinded to the individual group assignments for this study.

### Procedures

All participants received a mailed Pico G2 4K all-in-one head-mounted VR device at no cost. The on-demand, easy-to-use, and commercially available Pico G2 4K device has a 3840×2160 screen, a 72 FPS frame rate, and minimal visual latency. Although the treatment content differed between the EaseVRx and sham VR devices, all packaging and directions were identical. Participants were given access to online instructional materials for their headset.

Participants were instructed to complete 1 VR program session daily for the treatment duration. Study staff monitored device use and sent reminders as needed for survey completion. At end-of-treatment, staff managed the postage-paid return of the devices. Posttreatment study staff interaction was limited to survey completion reminders and responses to participant inquiries.

Compensation included US $6 per survey during and after treatment ($150 possible; prorated; received as Amazon eGift cards). Participants who completed ≥16 study surveys during treatment were eligible to receive a VR headset after study completion (n=73).

#### Therapeutic VR (EaseVRx)

**Figure 2:**
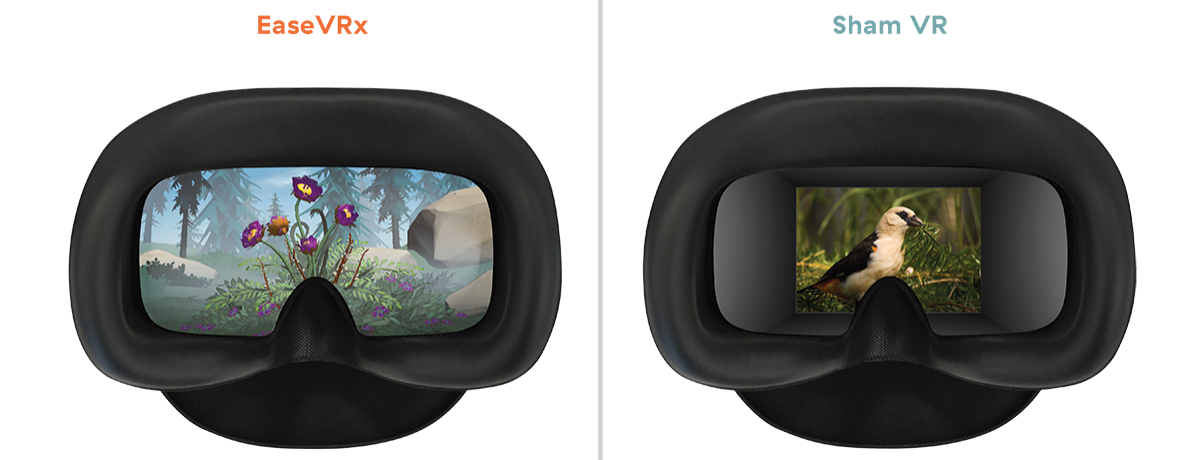
Visual display of EaseVRx (skills-based, interactive, 3D) and sham VR (noninteractive, 2D nature scenes). VR: virtual reality.

EaseVRx is a proprietary immersive, multimodal, skills-based, pain self-management VR program. EaseVRx incorporates evidence-based self-regulatory skills used in cognitive behavioral therapy for chronic pain (diaphragmatic breathing, biofeedback elements, cognition, and emotion regulation), mindfulness principles, and pain education into a multimodal therapeutic journey. The EaseVRx content is agnostic to pain type, condition, or disease (Figure 2).

The standardized 56-day program delivers the VR content through a prescribed sequence of daily immersive experiences grouped into 8 weekly themes relevant to living better with chronic pain. Content categories include pain education, relaxation and interoception, mindfulness escape, pain distraction games, and dynamic breathing. User exhalation is captured by an embedded microphone, providing interactive biodata-enabled therapeutics through synchrony with 3D visual displays and auditory feedback. VR sessions range from 2 to 16 minutes (average 6 minutes). Module content was designed to minimize emotional distress and cybersickness.

#### Sham VR

In compliance with VR-CORE clinical trial guidelines, we used an active and rigorous placebo comprised of nonimmersive 2D visual content [8]. Content included 20 rotating nature videos overlaid with music that was not relaxing, aversive, or distracting; content was devoid of pain education or pain management skills training (Figure 2). The average session duration closely matched that of EaseVRx.

### Data Collection and Timepoints

Data were collected through REDCap Cloud for patient-reported outcomes at pretreatment, end-of-treatment, and posttreatment months 1, 2, 3, and 6.

The baseline survey included demographic variables and a battery of outcome measures. Demographic variables included age, gender, education level, race, ethnicity, employment status, annual household income, relationship status, back pain duration, state of residence, and zip code. The baseline survey included other measures of secondary outcomes that were omitted from our prior publication [7] and this report because we found no significant between-group changes for these measures at end-of-treatment when treatment effects are most pronounced (items from the Pain Catastrophizing Scale [9], 2-item Pain Self-Efficacy Questionnaire [10], and 8-item Chronic Pain Acceptance Questionnaire [11], and self-reported prescription opioid and over-the-counter analgesic medication use).

### Measures

The Defense and Veterans Pain Rating Scale (DVPRS) [12] measured average pain intensity over the previous 24 hours using an 11-point numeric rating scale (0=no pain; 10=as bad as it could be and nothing else matters).

The DVPRS interference scale (DVPRS-II) [12] measured pain-related interference with activity, sleep, mood, and stress over the previous 24 hours (0=does not interfere; 10=completely interferes).

The National Institutes of Health (NIH) Patient-Reported Outcome Measurement Information System (PROMIS) short-form assessed physical function (version 6b) [13] and sleep disturbance (version 6a) [14] over the previous 7 days. The manuals’ conversion tables were used to calculate individual short-form T scores using item response theory algorithms [15]. T scores were computed for individual response patterns using the Bayesian expected a posteriori method [15,16].

### Adverse Event Monitoring

Participants were encouraged to contact staff about any problems with their device or treatment. Cybersickness was intended to be assessed immediately after treatment, but due to an error with the electronic survey, it was not captured until 1 month posttreatment.

### Statistical Analyses

All analyses involved 2-sided hypothesis tests, with α=.05, and were adjusted for multiple comparisons within the family of tests as appropriate. Group equivalence was assessed through univariate tests of association between groups (EaseVRx/sham VR) for all baseline demographic and clinical variables, with the chi-square and Kruskal-Wallis tests applied as appropriate.

The intent-to-treat data were analyzed in a mixed-model framework (PROC GLIMMIX in SAS 9.4M6) using a marginal (population-averaged) model to allow for correlated responses across repeated measures. Explanatory factors included treatment group, time, and time × treatment group. Treatment group (EaseVRx/sham VR) was specified as a fixed-effects factor. Time (pretreatment, end-of-treatment, and posttreatment months 1, 2, 3, and 6) was specified as a random-effects factor to allow for correlated responses using heterogeneous compound symmetry for the covariance structure within time. Analyses were conducted to assess (1) efficacy of treatment relative to pretreatment and (2) durability of treatment effects (end-of-treatment to month 6). Both analyses examined (1) EaseVRx vs sham VR between-group comparison across all timepoints and (2) whether the treatment group influenced the trajectory of the key variables over time. Efficacy, which included all 6 timepoints, was evidenced by significant treatment and time × treatment effects. We report multiplicity-adjusted Hochberg *P* values. Durability analyses were limited to end-of-treatment and posttreatment months 1, 2, 3, and 6. Durability was evidenced by a significant treatment effect but lack of time × treatment interaction, indicating sustained differences.

Missing values were not imputed for estimation of effects, but the predicted means were used in the graphical description. Linear mixed models were used as between-subject factors, and time of measurement was used as a within-subject factor. Effect sizes for the EaseVRx vs sham VR between-group comparison used the standardized mean difference version of Cohen *d* [17].

For each outcome variable, the effect size of the change pretreatment to 6 months posttreatment was assessed by treatment group using a repeated measures variation of Cohen *d* as *d_rm_* owing to the within-subject nature of the comparison [17]. We applied common effect size thresholds of 0.3 (small), 0.5 (medium), and 0.8 (large). Clinical meaningfulness of the change in each outcome variable was further assessed by calculating the mean percent improvement from pretreatment to 6 months posttreatment and applying Initiative on Methods, Measurement, and Pain Assessment in Clinical Trials (IMMPACT)-recommended thresholds of magnitude for moderate (30%) and substantial (50%) clinical importance [18].

Participant blinding was assessed by the proportion of participants in each group who correctly determined their treatment assignment.

To test the feasibility of home-based VR in individuals with lower SES [19], we assessed therapeutic VR treatment engagement (total duration of treatment and number of sessions) in participants with lower SES (defined as ≤high school education or ≤US $59,999 median annual household income) vs higher SES (defined as >high school education or ≥US $60,000 median annual household income; US $60,000 was the selected threshold because it is below the US median household income of US $67,000) [20].

## Results

### Overview

**Figure 3:**
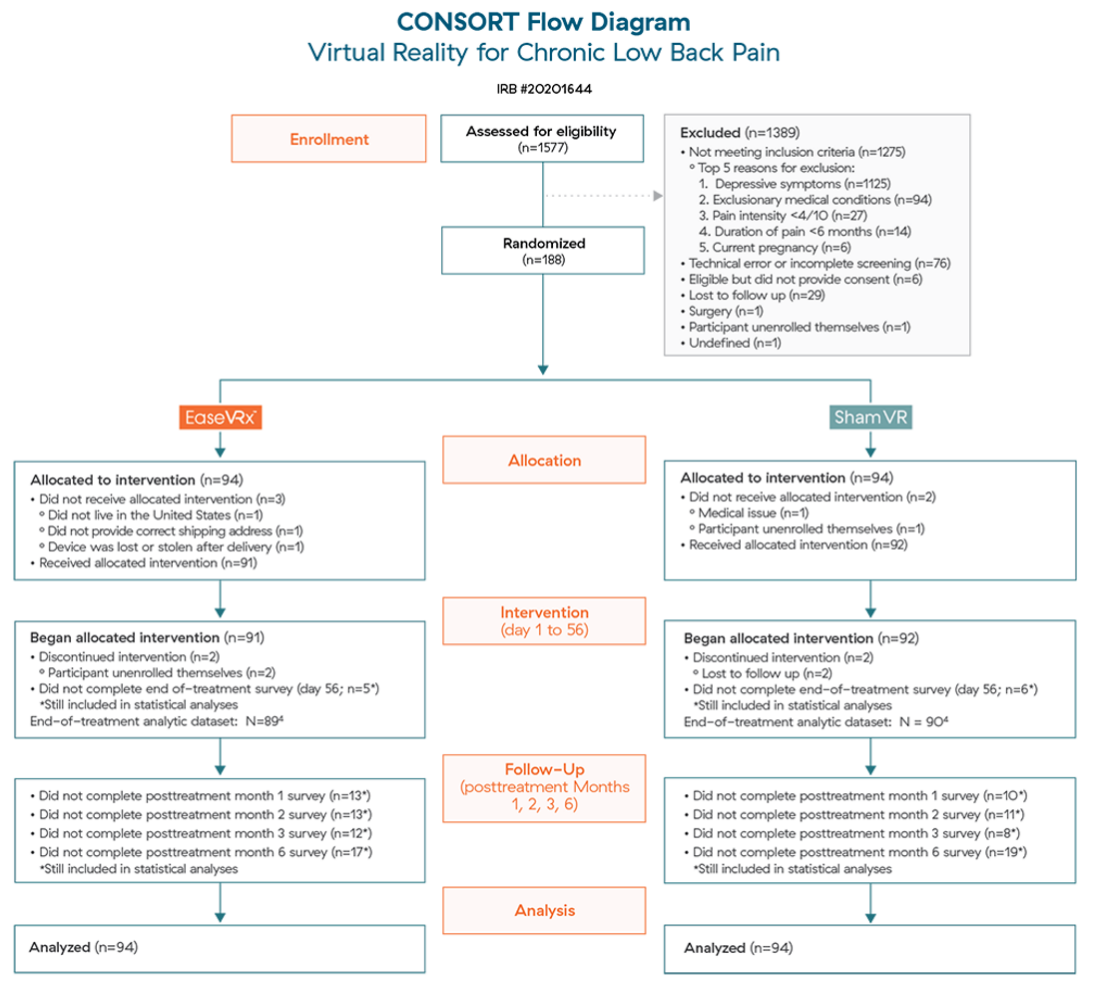
CONSORT flow diagram. VR: virtual reality.

Recruitment took place from July 6, 2020, to July 30, 2020. Of 1577 individuals who completed an online eligibility screener, 1389 were excluded primarily for meeting or exceeding the threshold for depressive symptoms (see Figure 3 for the CONSORT diagram). In total, 188 individuals were enrolled, randomized, and allocated to a treatment group. All participants were included in the dataset regardless of treatment engagement or survey completion at posttreatment months 1, 2, 3, and 6. Previously reported device-use data revealed nonsignificant between-group differences for treatment engagement [4]. Posttreatment survey completion rates were 83% (n=156) for month 1, 82% (n=155) for month 2, 85% (n=159) for month 3, and 74% (n=139) for month 6.

Table 1 displays baseline demographics and clinical characteristics. Table 2 displays pretreatment outcome variables for each group. The sample included participants from 40 US states. The sample was predominantly female (145/188, 77.1%) and Caucasian (171/188, 91.0%), with most participants having at least some college education (171/188, 91.0%). The mean age was 51.7 years (SD 13.2 years; range 18-81 years), and the mean duration of CLBP was ≥5 years.

**Table 1:** Baseline demographic and clinical characteristics by treatment group.

Variable		EaseVRx (n=94)	Sham VR^a^ (n=94)
**Gender, n (%)**			
	Male	23 (24)	20 (21)
	Female	71 (76)	73 (78)
	Other	0 (0)	1 (1)
Age (years), mean (SD)		52.1 (13.5)	51.3 (12.9)
Age range (years)		18.0-81.0	25.0-81.0
Age (years), median (IQR: Q1-Q3)		51.0 (41.0-62.0)	54.0 (41.0-62.0)
**Race, n (%)**			
	Asian	2 (2)	1 (1)
	Caucasian	82 (88)	88 (95)
	African American	5 (5)	1 (1)
	Multi-racial	2 (2)	3 (3)
	Other	2 (2)	0 (0)
	Missing	1 (1)	1 (1)
**Education, n (%)**			
	High school graduate	6 (6)	10 (11)
	Some college	22 (23)	17 (18)
	Associate	10 (11)	16 (17)
	Undergraduate	19 (20)	26 (28)
	Postgraduate	37 (39)	24 (26)
	Missing	0 (0)	1 (1)
**Employment, n (%)**			
	Part time	9 (10)	7 (8)
	Full time	39 (41)	36 (39)
	Not working	13 (14)	11 (12)
	Retired	17 (18)	21 (23)
	Unable to work	16 (17)	18 (19)
	Missing	0 (0)	1 (1)
**Income, n (%)**			
	Less than US $40,000	25 (27)	24 (26)
	US $40,000 to $59,999	24 (26)	19 (20)
	US $60,000 to $79,999	16 (17)	19 (20)
	Greater than US $80,000	28 (30)	32 (34)
	Missing	1 (1)	0 (0)
**Relationship, n (%)**			
	Married/civil union	55 (59)	63 (67)
	Divorced/widowed/separated	21 (23)	14 (15)
	Single	11 (12)	12 (13)
	Single-cohabitating	6 (6)	5 (5)
	Missing	1 (1)	0 (0)
**Pain duration, n (%)**			
	<1 year	7 (7)	1 (1)
	1 year to <5 years	25 (27)	26 (28)
	5 years to <10 years	17 (18)	25 (27)
	>10 years	45 (48)	42 (45)

^a^VR: virtual reality.

**Table 2:** Baseline outcome variables by treatment group.

Variable			EaseVRx (n=94)		Sham VR^a^ (n=94)		*P* value^b^
**Average pain intensity score**							.61
	Mean (SD)	5.1 (1.2)		5.2 (1.1)			
	Range	2.2-8.2		2.8-8.0			
	Median (IQR: Q1-Q3)	5.0 (4.2-5.8)		5.2 (4.4-5.8)			
**Pain-related activity interference score**							.43
	Mean (SD)	5.3 (1.8)		5.5 (1.5)			
	Range	1.2-10.0		1.0-8.8			
	Median (IQR: Q1-Q3)	5.6 (4.0-6.6)		5.6 (4.6-6.3)			
**Pain-related mood interference score**							.27
	Mean (SD)	4.4 (2.2)		4.7 (2.0)			
	Range	0.0-8.8		0.2-9.6			
	Median (IQR: Q1-Q3)	4.3 (2.8-5.8)		4.6 (3.4-6.0)			
**Pain-related sleep interference score**							.25
	Mean (SD)	4.8 (2.6)		5.3 (1.9)			
	Range	0.0-10.0		0.6-9.6			
	Median (IQR: Q1-Q3)	5.0 (3.0-7.0)		5.4 (4.0-6.4)			
**Pain-related stress interference score**							.76
	Mean (SD)	4.6 (2.2)		4.8 (2.0)			
	Range	0.0-10.0		0.6-9.6			
	Median (IQR: Q1-Q3)	4.7 (3.0-6.4)		5.0 (3.4-6.2)			
**PROMIS^c^ physical function score**							.30
	Mean (SD)	38.1 (5.1)		37.5 (4.7)			
	Range	21.0-48.9		27.1-59.0			
	Median (IQR: Q1-Q3)	37.6 (34.2-41.2)		37.6 (34.2-40.2)			
**PROMIS sleep disturbance score**							.17
	Mean (SD)	56.7 (5.2)		57.7 (4.3)			
	Range	44.2-67.5		45.5-69.0			
	Median (IQR: Q1-Q3)	56.3 (53.3-60.4)		58.3 (55.3-60.4)			

^a^VR: virtual reality.

^b^Kruskal-Wallis *P* value.

^c^PROMIS: Patient-Reported Outcome Measurement Information System.

### Primary Outcomes

**Figure 4:**
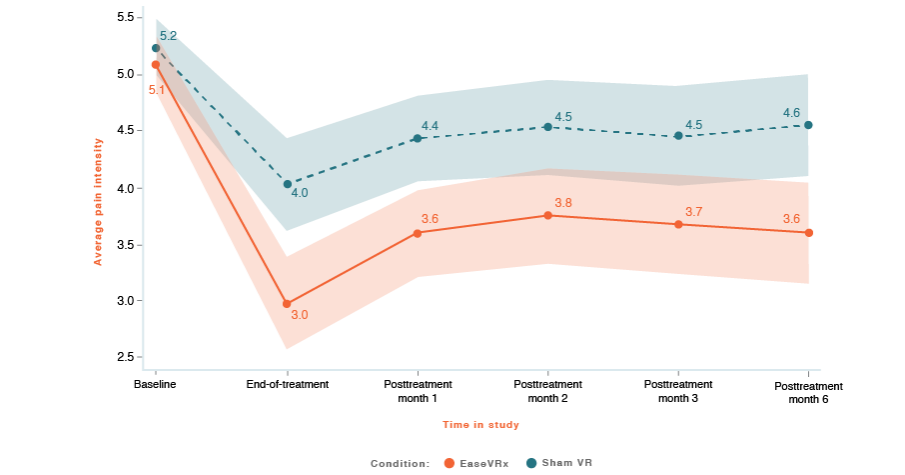
Average pain intensity. The color bands represent 95% CI for the mean after correcting for multiple comparisons. Overlapping bands indicate nonsignificant group differences of simple main effects within each timepoint. VR: virtual reality.

**Figure 5:**
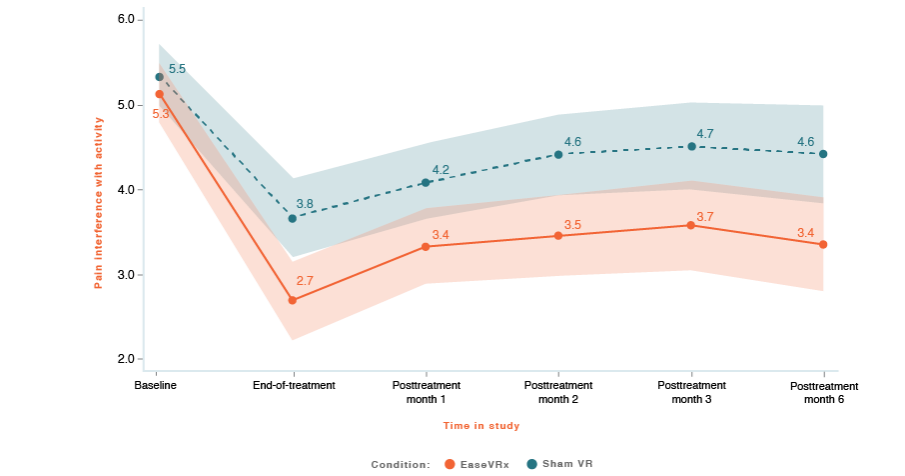
Pain interference with activity. The color bands represent 95% CI for the mean after correcting for multiple comparisons. Overlapping bands indicate nonsignificant group differences of simple main effects within each timepoint. VR: virtual reality.

**Figure 6:**
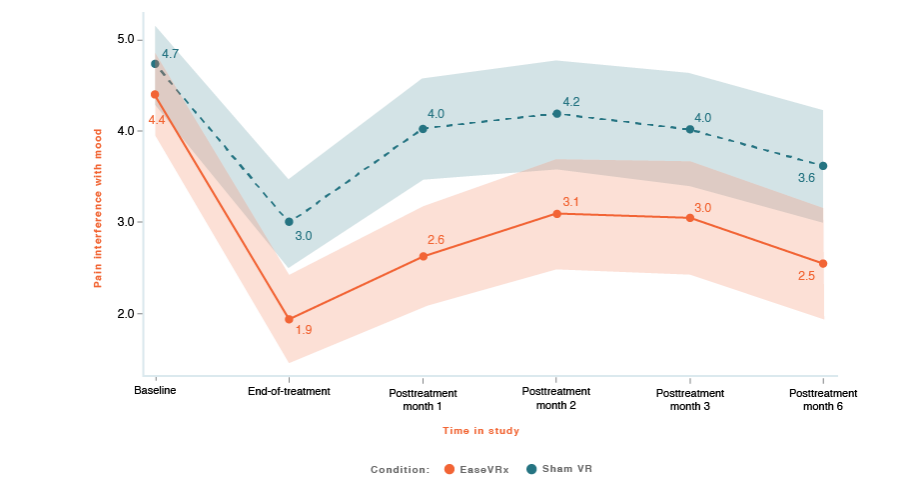
Pain interference with mood. The color bands represent 95% CI for the mean after correcting for multiple comparisons. Overlapping bands indicate nonsignificant group differences of simple main effects within each timepoint. VR: virtual reality.

**Figure 7:**
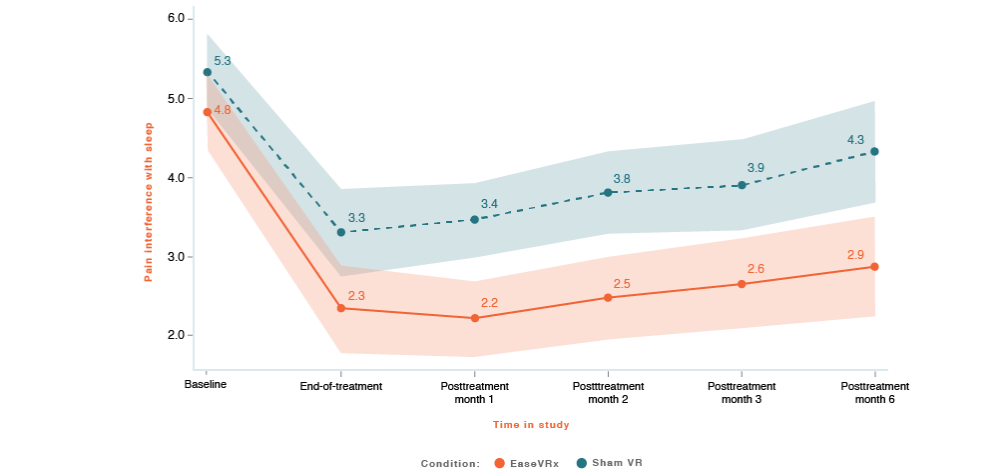
Pain interference with sleep. The color bands represent 95% CI for the mean after correcting for multiple comparisons. Overlapping bands indicate nonsignificant group differences of simple main effects within each timepoint. VR: virtual reality.

**Figure 8:**
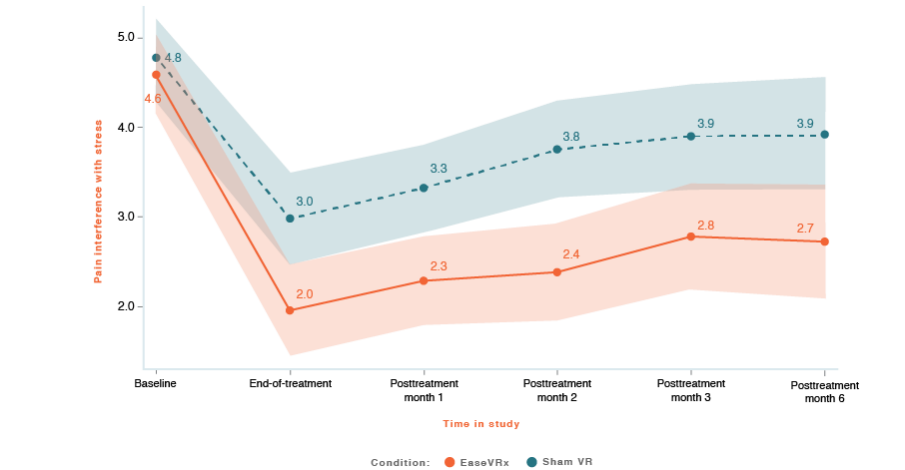
Pain interference with stress. The color bands represent 95% CI for the mean after correcting for multiple comparisons. Overlapping bands indicate nonsignificant group differences of simple main effects within each timepoint. VR: virtual reality.

**Figure 9:**
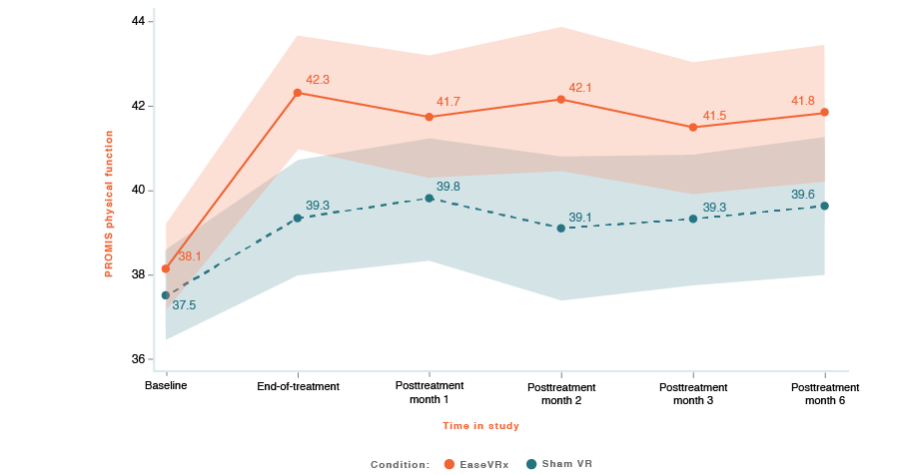
PROMIS physical function. The color bands represent 95% CI for the mean after correcting for multiple comparisons. Overlapping bands indicate nonsignificant group differences of simple main effects within each timepoint. PROMIS: Patient-Reported Outcome Measurement Information System; VR: virtual reality.

**Figure 10:**
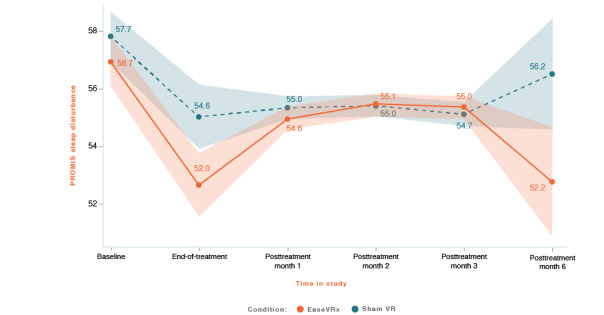
PROMIS sleep disturbance. The color bands represent 95% CI for the mean after correcting for multiple comparisons. Overlapping bands indicate nonsignificant group differences of simple main effects within each timepoint. PROMIS: Patient-Reported Outcome Measurement Information System; VR: virtual reality.

We applied the analytic plan outlined above to each primary outcome. For each primary outcome figure referenced below, the x-axis represents time and the color bands represent 95% CI for the mean after correcting for multiple comparisons (Tukey-Kramer). Overlapping bands indicate nonsignificant group differences (*P* values) of simple main effects within each timepoint. Table 3 includes the corresponding model effects for each primary outcome in Figures 4-10.

**Table 3:** Model effects for primary outcomes.

Factor		Numerator df^a^	Denominator df	*F* value	*P* value
**Pain intensity**					
	Treatment	1	186	11.05	.001
	Time	5	758	47.43	<.001
	Time × treatment	5	758	4.05	.001
**Pain interference with activity**					
	Treatment	1	186	9.16	.003
	Time	5	758	56.77	<.001
	Time × treatment	5	758	2.95	.001
**Pain interference with mood**					
	Treatment	1	186	10.59	.001
	Time	5	758	35.66	<.001
	Time × treatment	5	758	2.07	.07
**Pain interference with sleep**					
	Treatment	1	186	13.82	<.001
	Time	5	758	49.71	<.001
	Time × treatment	5	758	1.84	.10
**Pain interference with stress**					
	Treatment	1	186	10.23	.002
	Time	5	758	46.94	<.001
	Time × treatment	5	758	3.34	.006
**PROMIS^b^ physical function**					
	Treatment	1	186	5.57	.02
	Time	5	758	22.78	<.001
	Time × treatment	5	758	2.92	.01
**PROMIS sleep disturbance**					
	Treatment	1	186	9.82	.002
	Time	5	758	14.68	<.001
	Time × treatment	5	758	3.78	.002

^a^df: degree of freedom.

^b^PROMIS: Patient-Reported Outcome Measurement Information System.

#### Pain Intensity

Average pain intensity was lower in the EaseVRx group than in the sham VR group (Cohen *d_s_*=0.48; *P*=.001). Both treatment groups had lower average pain intensity from pretreatment to 6 months posttreatment (*P*<.001). While there was no between-group difference at pretreatment, at end-of-treatment, EaseVRx participants indicated lower pain intensity relative to sham VR, and this difference was maintained at month 6 (*P*=.001; Hochberg *P*=.006 after multiplicity correction; see Figure 4).

For pain intensity at 6 months posttreatment, the mean percentage change was −31.3% (moderate clinical importance) for the EaseVRx group and −15.9% (minimal clinical importance) for the sham VR group. We found that 52.1% (37/71) of EaseVRx and 25.0% (17/68) of sham VR participants achieved the threshold for moderate clinical meaningfulness (≥30%) and 38.0% (27/71) and 13.2% (9/68), respectively, achieved the threshold for substantial clinical meaningfulness (≥50%).

To evaluate durability, we compared end-of-treatment with the 6-month follow-up. On average, pain intensity was lower in the EaseVRx group than in the sham VR group (*P*=.004). We observed a significant effect of time (*P*<.001) but not time × treatment for pain intensity, indicating sustained superiority of EaseVRx over sham VR through 6 months posttreatment.

#### Pain-Related Interference With Activity

Average pain-related interference with activity was lower in the EaseVRx group than in the sham VR group (Cohen *d_s_*=0.44; *P*=.003). Both groups had lower activity interference from pretreatment through month 6 (*P*<.001). Finally, we observed a pronounced between-group difference at end-of-treatment but not pretreatment (*P*=.01; Hochberg *P*=.04 after multiplicity correction; see Figure 5).

At 6 months posttreatment, the mean percentage change was −34.8% for the EaseVRx group and −20.8% for the sham VR group. We found that 60.6% (43/71) of EaseVRx and 39.7% (27/68) of sham VR participants achieved the threshold for moderate clinical meaningfulness and 50.7% (36/71) and 25.0% (17/68), respectively, achieved the threshold for substantial clinical meaningfulness.

Comparing end-of-treatment with the 6-month follow-up, pain-related interference with activity was lower in the EaseVRx group than in the sham VR group (*P*=.006). We observed a significant effect of time (*P*<.001) but not time × treatment (*P*=.92) for pain-related interference with activity, indicating sustained superiority of EaseVRx.

#### Pain-Related Interference With Mood

On average, pain-related interference with mood was lower in the EaseVRx group than in the sham VR group (Cohen *d_s_*=0.47; *P*=.001). Both groups had lower mood interference from pretreatment through month 6 (*P*<.001). The time × treatment effect was not significant (*P*=.07; Hochberg *P*=.10 after multiplicity correction; see Figure 6).

At 6 months posttreatment, the mean percentage change for pain-related interference with mood was −39.2% for EaseVRx and −25.3% for sham VR. We found that 59.2% (42/71) of EaseVRx and 48.5% (33/68) of sham VR participants achieved the threshold for moderate clinical meaningfulness and 54.9% (39/71) and 41.2% (28/68), respectively, achieved the threshold for substantial clinical meaningfulness.

To evaluate durability, we compared end-of-treatment with the 6-month follow-up. On average, pain-related interference with mood was lower in the EaseVRx group than in the sham VR group (*P*=.003). We observed a significant effect of time (*P*<.001) but not time × treatment (*P*=.79), indicating sustained superiority of EaseVRx over sham VR through 6 months posttreatment.

#### Pain-Related Interference With Sleep

On average, pain-related sleep interference was lower in the EaseVRx group than in the sham VR group (Cohen *d_s_*=0.54; *P*<.001). Both groups had lower sleep interference from pretreatment through month 6 (*P*<.001). The time × treatment effect was not significant (*P*=.10; Hochberg *P*=.10 after multiplicity correction; see Figure 7).

At 6 months posttreatment, the mean percentage change was −44.5% for the EaseVRx group and −18.9% for the sham VR group. We found that 63.4% (45/71) of EaseVRx and 45.6% (31/68) of sham VR participants achieved the threshold for moderate clinical meaningfulness and 47.9% (34/71) and 32.4% (22/68), respectively, achieved the threshold for substantial clinical meaningfulness.

Comparing end-of-treatment with the 6-month follow-up, pain-related interference with sleep was lower in the EaseVRx group than in the sham VR group (*P*<.001). We also observed a significant effect of time (*P*<.001) but not time × treatment (*P*=.89), indicating sustained superiority of EaseVRx over sham VR through 6 months posttreatment.

#### Pain-Related Interference With Stress

On average, pain-related stress interference was lower in the EaseVRx group than in the sham VR group (Cohen *d_s_*=0.47; *P*=.001). Both groups had lower pain-related stress interference from pretreatment through month 6 (*P*<.001). While there was no between-group difference at pretreatment, there was a pronounced difference at end-of-treatment and at month 6 (time × treatment *P*=.006; Hochberg *P*=.02 after multiplicity correction; see Figure 8).

At 6 months posttreatment, the mean percentage change in pain-related interference with stress was −42.5% for the EaseVRx group and −23.3% for the sham VR group. We found that 67.6% (48/71) of EaseVRx and 39.7% (27/68) of sham VR participants achieved the threshold for moderate clinical meaningfulness and 60.6% (43/71) and 30.9% (21/68), respectively, achieved the threshold for substantial clinical meaningfulness.

Comparing end-of-treatment with the 6-month follow-up, pain-related interference with stress was lower in the EaseVRx group than in the sham VR group (*P*=.002). We observed a significant effect of time (*P*<.001) but not time × treatment (*P*=.86), indicating sustained superiority of EaseVRx over sham VR through 6 months posttreatment.

### Secondary Outcomes

#### Physical Function

Physical function was higher in the EaseVRx group than in the sham VR group (Cohen *d_s_*=0.34; *P*=.02), and both groups demonstrated increased physical function from pretreatment to 6 months posttreatment (*P*<.001). While there was no between-group difference at pretreatment, a between-group difference was pronounced at end-of-treatment through 6 months posttreatment (time × treatment *P*=.01; see Figure 9).

At 6 months posttreatment, the mean improvement in physical function was 10.5% for the EaseVRx group and 5.9% for the sham VR group, with changes in both groups categorized as clinically unimportant. We found that 12.7% (9/71) of EaseVRx and 4.4% (3/68) of sham VR participants reached the moderate clinical meaningfulness threshold. For substantial clinical meaningfulness, 4.2% (3/71) of EaseVRx participants and no sham VR participants achieved the threshold.

Comparing end-of-treatment with the 6-month follow-up, physical function was higher in the EaseVRx group than in the sham VR group (*P*=.02). The level of physical function was maintained from end-of-treatment to month 6 (*P*=.77). The time × treatment interaction effect was not significant (*P*=.45), indicating a sustained end-of-treatment effect (albeit of negligible clinical importance) for EaseVRx vs sham VR.

#### Sleep Disturbance

Sleep disturbance was lower in the EaseVRx group than in the sham VR group (Cohen *d_s_*=0.46; *P*=.002). Both groups had decreased sleep disturbance over time (*P*<.001). While there was no between-group difference at pretreatment, at end-of-treatment, sleep disturbance was lower in the EaseVRx group than in the sham VR group, which did not sustain in posttreatment months 1, 2, 3, and 6 (time × treatment *P*=.002; see Figure 10).

At 6 months posttreatment, the mean percentage change in sleep disturbance was −8.8% for EaseVRx and −2.1% for sham VR. While 8.5% (6/71) of EaseVRx and 1.5% (1/68) of sham VR participants achieved the moderate clinical meaningfulness threshold, no EaseVRx or sham VR participants achieved the threshold for substantial clinical meaningfulness.

Comparing end-of-treatment with 6 months post-treatment, sleep disturbance was lower in the EaseVRx group than in the sham VR group (*P*=.002). There was a significant effect of time (*P*=.003). The end-of-treatment superiority of EaseVRx over sham VR for reduction in sleep disturbance was absent in posttreatment months 1, 2, and 3, and re-emerged at month 6 (time × treatment *P*=.002).

Assessing participant blinding 6 months posttreatment, 75% of EaseVRx and 71% of sham VR participants accurately identified their randomly assigned treatment. These proportions did not differ between groups (*P*>.05) and were significantly above chance.

Lower SES (n=97) and higher SES (n=91) participants were statistically equivalent for treatment engagement as indexed by the total duration of EaseVRx treatment time and total number of EaseVRx experiences.

## Discussion

This report describes the 6-month durability of treatment effects for a randomized placebo-controlled trial of an 8-week self-administered skills-based VR program (EaseVRx) compared with a sham VR program in adults with CLBP. Intention-to-treat analysis performed on data collected 6 months after treatment revealed some regression to the mean with continued superiority of therapeutic EaseVRx over sham VR for reductions in pain intensity and pain-related interference (activity, stress, and sleep). Six-month posttreatment results exceeded thresholds for clinical meaningfulness, with effect sizes ranging from 0.34 to 0.54. Between-group differences for physical function and sleep disturbance at 6 months were statistically significant but not clinically meaningful. Combined, the results support the 6-month analgesic efficacy of a fully automated, 8-week, home-based VR program for CLBP. Recent meta-analyses of VR noted a lack of high-quality efficacy studies for chronic pain [21], except for those involving physical rehabilitation programs [22]. To our knowledge, our investigations on the extended efficacy of VR are the first involving home-based pain management without physical rehabilitation.

Findings from this study further support the efficacy of home-based VR treatment and may inform clinician and patient expectations, reimbursement models, and prescription pathways for CLBP. Critics have questioned whether participant education or socioeconomic factors might predict user engagement. Accordingly, we examined whether participant education level (high school level or less vs at least some college education) or household annual income (above vs below the US median) as a composite metric of SES would impact treatment engagement. While our examination of the impact of SES on user engagement is preliminary and may be subject to selection bias, we found equivalent engagement between lower and higher SES individuals with EaseVRx. These data potentially refute a perception that a high-tech digital treatment, such as VR, may be infeasible in lower SES individuals, and suggest that digital therapeutics, like EaseVRx, represent an opportunity to reach CLBP patients in historically underserved areas. These data also align with our published EaseVRx usability ratings, in which this study sample indicated that the device was as easy to use as an iPhone [4].

Key strengths of this study include (1) randomized placebo-controlled design; (2) intention-to-treat analyses; (3) correction for multiplicity; (4) longitudinal design and data collection to 6 months posttreatment; and (5) participant blinding to treatment group.

Our findings should be placed in the context of several limitations. First, the study sample had low levels of depressive symptoms and was specific to CLBP. The sample was also mainly female and white, and had some college education, thus limiting the generalizability to the broader population. The study relied only on participant-reported data and no objective data on medical or mental health conditions or receipt of additional pain treatments during the study period. The 26% attrition rate at 6 months was similar between treatment groups, and its effects were mitigated by the intention-to-treat analytic approach. Finally, at the end of the study roughly 73% of the sample correctly guessed their treatment group assignment, suggesting that, despite extensive efforts to maintain face validity of sham VR and following published guidance [8], the actual blinding failed. Despite this, we previously reported equivalent treatment engagement between both groups and symptom benefits gained by sham VR participants [7], albeit sham VR was substantially less efficacious than therapeutic VR. Nevertheless, equivalent engagement in sham VR suggests acceptable control in terms of time and attention, exposure to treatment, device use, survey completion, and participant compensation.

Placebo effects are well known in clinical studies [23]. The clinically meaningful efficacy of open-label placebo supports our finding of sham VR benefits even when treatment group assignment is correctly guessed [24]. The superiority and durability of the therapeutic response to therapeutic immersive VR is even more intriguing in the context of placebo interference with analgesic outcomes.

In-progress research includes an active national pragmatic effectiveness study designed to ascertain the long-term treatment effects of therapeutic VR in patients with CLBP who are highly diverse in race, ethnicity, education level, and symptom profiles. Future research should extend efficacy investigations for home-based VR to other pain conditions and diagnoses, as well as examine mechanisms of treatment effects in real-world patient populations. Finally, while we previously reported very high treatment engagement rates for both study groups, future research may investigate the characteristics and needs of individuals who exhibit lower engagement rates, nonresponsiveness, or higher rates of regression to the mean over time, and develop strategies to optimize outcomes for these subpopulations.

The 6-month durability of clinically meaningful reductions in pain intensity and pain-related interference suggests that this effective digital therapeutic approach may transcend many current barriers and improve patient access to effective nonpharmacologic pain care for CLBP.

## Abbreviations

CLBP: chronic low back pain
DVPRS: Defense and Veterans Pain Rating Scale
PROMIS: Patient-Reported Outcome Measurement Information System
SES: socioeconomic status
VR: virtual reality

## Data Availability

A data dictionary and deidentified participant data will be made available after publication and upon approved request of a detailed meta-analytic study proposal. Requests should be made to the corresponding author along with a study proposal and a signed data access agreement.
