# Correction: Durability of the Treatment Effects of an 8-Week Self-administered Home-Based Virtual Reality Program for Chronic Low Back Pain: 6-Month Follow-up Study of a Randomized Clinical Trial

**DOI:** 10.2196/40038

**Published:** 2022-06-08

**Authors:** Laura Garcia, Brandon Birckhead, Parthasarathy Krishnamurthy, Ian Mackey, Josh Sackman, Vafi Salmasi, Robert Louis, Carina Castro, Roselani Maddox, Todd Maddox, Beth D Darnall

**Affiliations:** 1 AppliedVR Van Nuys, CA United States; 2 Johns Hopkins School of Medicine Baltimore, MD United States; 3 University of Houston Houston, TX United States; 4 Stanford University School of Medicine Palo Alto, CA United States; 5 Hoag Memorial Hospital Newport Beach, CA United States; 6 Stanford School of Medicine Palo Alto, CA United States

In “Durability of the Treatment Effects of an 8-Week Self-administered Home-Based Virtual Reality Program for Chronic Low Back Pain: Follow-up Study of a Randomized Clinical Trial” (J Med Internet Res 2022;24(5):e37480) the authors made one clarification.

In the originally published paper, the title appeared as follows:

“Durability of the Treatment Effects of an 8-Week Self-administered Home-Based Virtual Reality Program for Chronic Low Back Pain: Follow-up Study of a Randomized Clinical Trial”

In the corrected version of the paper, the title has been changed to:

“Durability of the Treatment Effects of an 8-Week Self-administered Home-Based Virtual Reality Program for Chronic Low Back Pain: 6-Month Follow-up Study of a Randomized Clinical Trial”

The correction will appear in the online version of the paper on the JMIR Publications website on June 8, 2022 together with the publication of this correction notice. Because this was made after submission to PubMed, PubMed Central, and other full-text repositories, the corrected article has also been resubmitted to those repositories.

